# Lung quantitative ultrasound to stage and monitor interstitial lung diseases

**DOI:** 10.1038/s41598-024-66390-6

**Published:** 2024-07-16

**Authors:** Azadeh Dashti, Roshan Roshankhah, Theresa Lye, John Blackwell, Stephanie Montgomery, Thomas Egan, Jonathan Mamou, Marie Muller

**Affiliations:** 1 Department of Mechanical and Aerospace Engineering, North Carolina State University, Raleigh, NC 27695 USA; 2 Topcon Advanced Biomedical Imaging Laboratory, Topcon Healthcare, Oakland, NJ 07436 USA; 3 Department of Radiology, Weill Cornell Medicine, New York, NY 10022 USA; 4 Department of Surgery, University of North Carolina at Chapel Hill, Chapel Hill, NC 27599 USA; 5 AKC Canine Health Foundation, Raleigh, NC 27617 USA; 6 Department of Biomedical Engineering, North Carolina State University, Raleigh, NC 27695 USA

**Keywords:** Diagnostic markers, Prognostic markers, Biomedical engineering, Preclinical research, Translational research

## Abstract

Chronic interstitial lung diseases (ILDs) require frequent point-of-care monitoring. X-ray-based methods lack resolution and are ionizing. Chest computerized tomographic (CT) scans are expensive and provide more radiation. Conventional ultrasound can detect severe lung damage via vertical artifacts (B-lines). However, this information is not quantitative, and the appearance of B-lines is operator- and system-dependent. Here we demonstrate novel ultrasound-based biomarkers to assess severity of ILDs. Lung alveoli scatter ultrasound waves, leading to a complex acoustic signature, which is affected by changes in alveolar density due to ILDs. We exploit ultrasound scattering in the lung and combine quantitative ultrasound (QUS) parameters, to develop ultrasound-based biomarkers that significantly correlate (*p* = 1e^−4^ for edema and *p* = 3e^−7^ for fibrosis) to the severity of pulmonary fibrosis and edema in rodent lungs. These innovative QUS biomarkers will be very significant for monitoring severity of chronic ILDs and response to treatment, especially in this new era of miniaturized and highly portable ultrasound devices.

## Introduction

Lung ultrasound (LUS) remains challenging because of multiple scattering of ultrasound waves by air-filled alveoli that prevents creating an image. Conventional ultrasound imaging is based on the assumption of a known speed of sound in tissue. In highly heterogeneous media such as lung tissue, ultrasound waves encounter numerous reflections and scattering events, leading to complex propagation paths. This prevents obtaining images that accurately represent lung structures using conventional ultrasound.

Although conventional LUS imaging does not provide detailed structural information about the parenchyma, physicians have used it to detect signs of potential lung damage by observing artifacts in LUS images. Efforts have been made to standardize the interpretation of these artifacts, such as vertical and horizontal artifacts known as B-lines and A-lines, respectively. For example, B-lines in LUS images have been utilized to detect the presence of pulmonary fibrosis^1^. The appearance of these artifacts has also been shown to correlate with increased extravascular lung water^2^, interstitial lung diseases (ILDs)^2–4^, cardiogenic and non-cardiogenic lung edema^5^, interstitial pneumonia^6^, and lung contusion^7^. The physical origin of these artifacts is commonly attributed to ultrasound beam reverberations in acoustical traps made of poorly aerated regions^8^.

However, interpreting these artifacts is highly subjective since they are qualitative and dependent on imaging parameters such as transmit ultrasound frequency^9,10^. It is possible that evaluating this frequency dependence using spectroscopic methods could lead to an increased specificity of lung ultrasound^10^. However, spectroscopic ultrasound is not currently available with conventional LUS, and exploiting B-lines remains qualitative. Nevertheless, this study was one of the first to report results obtained in vivo in humans.

Consequently, the effectiveness of diagnosing and monitoring ILDs using conventional LUS is not possible. There is a clear need for a quantitative approach to assess lung parenchymal microstructure. This would enable monitoring severity of some ILDs, especially in point-of-care settings. The rationale for the present study is to leverage this purported weakness of LUS: we exploit scattering as a source of contrast for characterizing lung tissue. Our novel hypothesis is that ILD-induced changes in alveolar structure related to interstitial lung diseases will lead to altered scattering patterns and acoustic signatures that can be exploited to quantify these changes^11^.

We quantify and characterize this acoustic signature by using four families of quantitative ultrasound (QUS) parameters, related to (1) the relative contributions of multiple and single scattering (MS and SS), (2) the backscatter coefficient (BSC), (3) envelope statistics (EnvS) and (4) attenuation (Att). The output of these methods (i.e., the lung QUS (LQUS) parameters) effectively quantifies severity of pulmonary edema and fibrosis because each of these LQUS parameters is a function of the scattering properties of lung tissue that is related to lung microstructure. Pulmonary diseases all affect the parenchymal microstructure in unique ways. Since alveoli act as scatterers, LQUS parameters will reflect parenchymal changes such as alveolar density and size, alveolar flooding, alveolar wall thickness, or spatial heterogeneity. Measuring a variety of LQUS parameters is critical to ensure specificity of the method, because we expect different combinations of these LQUS parameters to be sensitive to different ILDs. This study shows that different combinations of LQUS parameters enable creation of two different, easy-to-interpret LQUS scores that are respectively sensitive and specific to pulmonary fibrosis and pulmonary edema.

## Results

### Raw ultrasound data in rodent models of lung edema and fibrosis

We used a surgical model of ischemia–reperfusion injury to create various degrees of pulmonary edema in 18 Sprague–Dawley rats. We used a pharmaceutical model of bleomycin inhalation to create various degrees of pulmonary fibrosis in 24 Sprague Dawley rats. Among the fibrotic rats, six were administered Nintedanib, to demonstrate the impact of treatment. Twelve healthy untreated animals were used as controls (six for edema and six for fibrosis). Raw ultrasound data were acquired using a fully programmable ultrasound research scanner (Verasonics, Kirkland, WA). A 128-element linear array transducer (L11-5v, Verasonics), with a central frequency of 7.8 MHz was placed directly on the lung surface using ultrasound coupling gel. After ultrasound data acquisition, the animals were euthanized, and the lungs were excised. Pulmonary edema severity was quantified by the wet/dry weight ratio (W/D)^12^. Pulmonary fibrosis severity was quantified histologically by a board-certified veterinary pathologist using the modified Ashcroft score^13,14^.

### Quantitative ultrasound biomarkers of pulmonary edema and fibrosis

**Figure 1:**
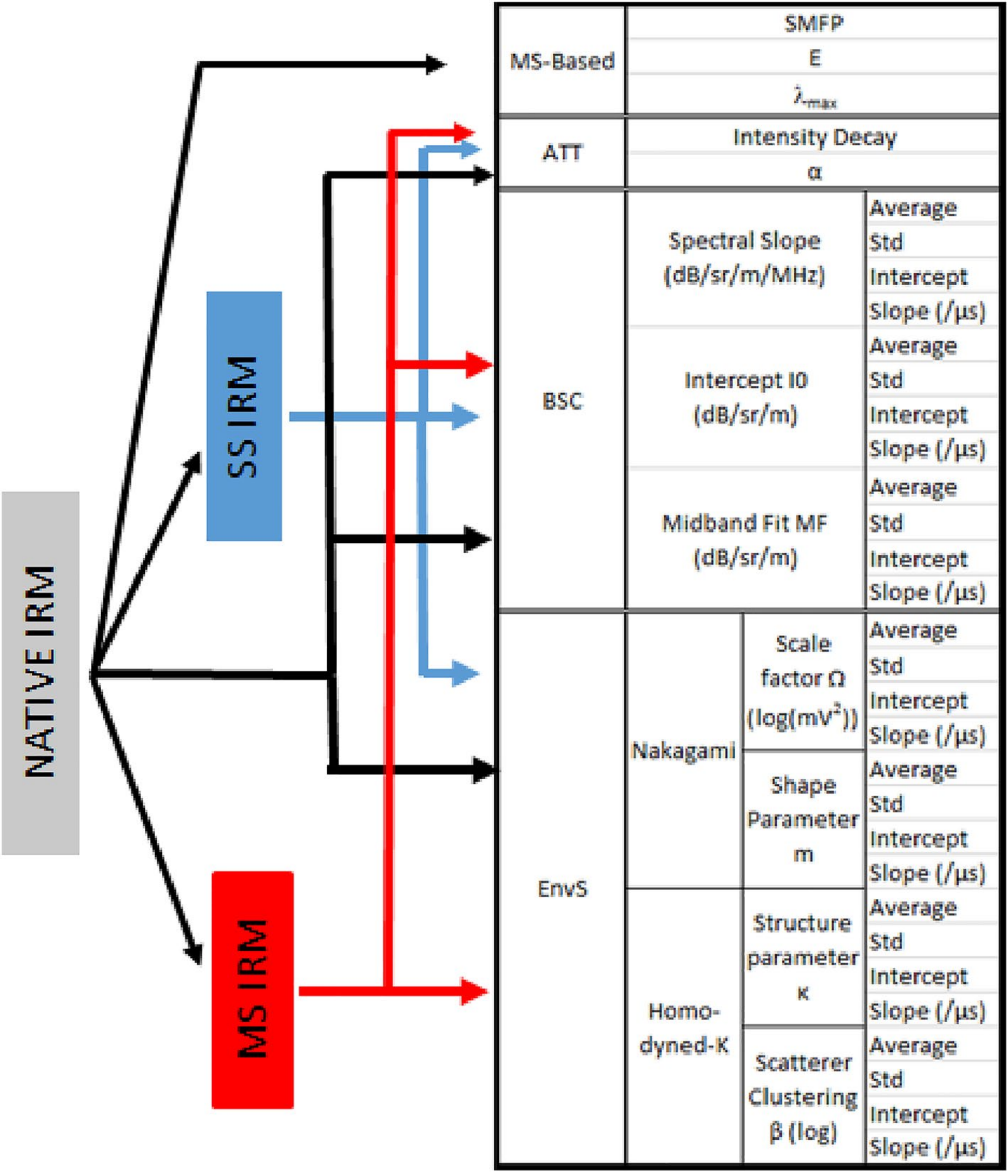
Summary of QUS parameters extracted from a single data set, ie a single Inter-element response matrix (IRM).

Raw ultrasound data were processed in order to measure LQUS parameters from four families of methods; Fig. 1 summarizes all the LQUS parameters that were obtained from a single ultrasound data set.

**Figure 2:**
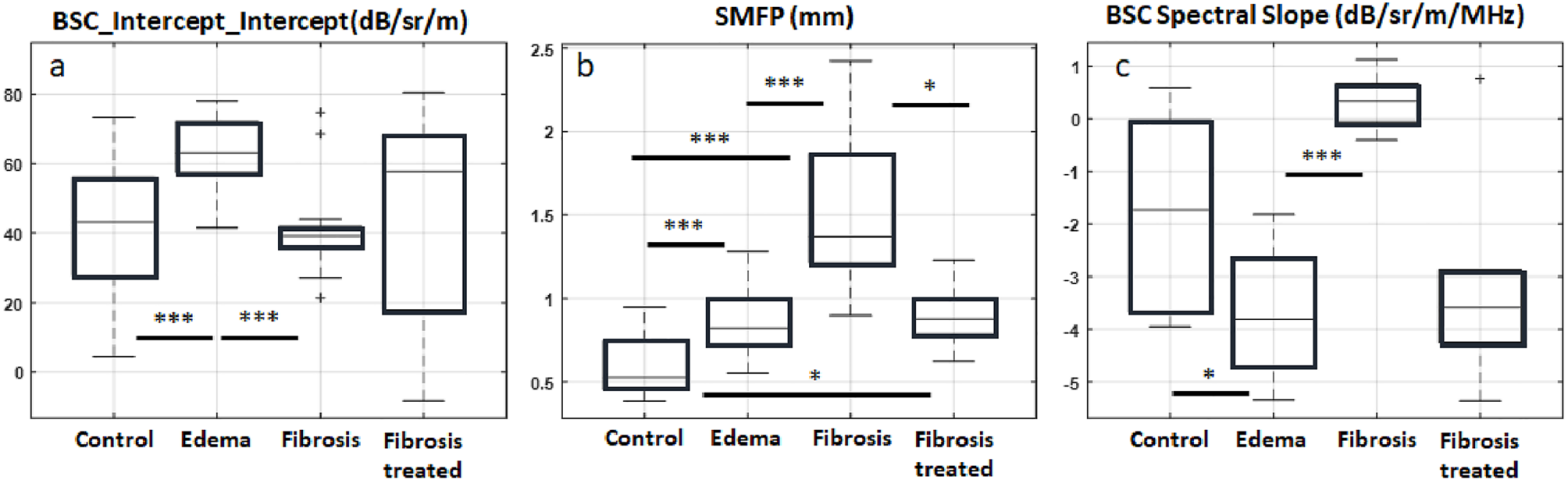
(**a**) Significant differences are observed between the Intercept of the BSC Intercept for control and edematous lungs (*p* = 3.4 * 10^–4^). (**b**) Significant differences are observed between Scattering Mean Free Path (SMFP) for control and fibrotic lungs (Bonferroni corrected *p*′ = 1.3 * 10^–6^). The SMFP also shows slower progression of fibrosis in the group of animals treated with Nintedanib compared to the fibrotic group (Bonferroni corrected *p*′ = 1.9 * 10^−2^). (**c**) The BSC Spectral slope allowed to discriminate between control and fibrosis (*p* < 10^−15^). *Indicates a *p* value lower than 0.05. **Indicates a *p* value lower than 0.01, ***indicates a *p *value lower than 0.001.

Multiple LQUS parameters showed significant differences between the edematous group and the control group, between the fibrotic group and the control group, and between the fibrotic and the edematous group (Fig. 2). For example, the Intercept of the BSC Intercept was on average equal to 40.9 ± 10.5 dB/sr/m in the control group and 64.0 ± 4.8 dB/sr/m in the edematous group. The Intercept of the BSC Intercept showed significant differences between these two groups (*p* = 3.4 * 10^–4^), and between the fibrosis and edema groups (*p* = 2.7 * 10^–7^), but not between the control and fibrosis groups (Fig. 2a). As another example of a measured LQUS parameter, the Scattering Mean Free Path (SMFP) represents the average distance between scatterers. It is expected to increase with fibrosis, due to increasing inter-alveolar distances. In the control group, the SMFP was found to be 0.60 ± 0.09 mm. In the fibrotic group, the SMFP was 1.43 ± 0.24 mm. In the fibrotic group treated with Nintedanib, the SMFP was 0.94 ± 0.08 mm, falling between the control and the fibrotic group. ANOVA demonstrated differences in SMFP between the control, fibrotic and treated groups (*p* = 1.3 * 10^–6^) as well as between the fibrosis and Nintedanib-treated groups (*p* = 1.9 * 10^–2^) (Fig. 2b). Multiple LQUS parameters allowed discrimination between the fibrosis and edema groups, including the SMFP (*p* = 3.5 * 10^–5^) and the BSC Spectral Slope, as shown in Fig. 2b and c. BSC Spectral Slope was on average equal to 0.35 ± 0.23 dB/sr/m/MHz in the edema group, and equal to − 3.73 ± 0.56 dB/sr/m/MHz in the fibrosis group. Through an unpaired t-test, BSC Intercept was found significantly different in the edema and fibrosis groups (*p* < 10^–15^).

### Sensitivity to the severity of edema and fibrosis

Pearson linear correlations between each LQUS parameter and the fibrosis histology score or the W/D were computed. Overall, 29 LQUS parameters were significantly correlated (i.e., *p* < 0.05) to the W/D ratio in the edema study, and 23 LQUS were significantly correlated to the histology score in the fibrosis study. The LQUS parameters yielding the four strongest correlations are listed in Table 1.

**Table 1:** Left: Most significant linear correlations between single LQUS parameters and severity of edema as measured by W/D. Right: Most significant correlations between single LQUS parameters and severity of fibrosis as measured by histology.

EDEMA (correlation with W/D)			FIBROSIS (correlation with histology)		
Parameter	R	*p*	Parameter	R	*p*
MF_slope	0.61	1.70E−03	MF_Intercept	0.6	2.10E−03
Nakagami m Avg	0.59	2.70E−03	SS_IDR	− 0.59	2.40E−03
MF_Std	− 0.58	2.70E−03	Nakagami m Intercept	− 0.57	4.00E−03
Nakagami Ω Slope	0.58	2.80E−03	Spectral Slope	0.51	1.10E−02

**Figure 3:**
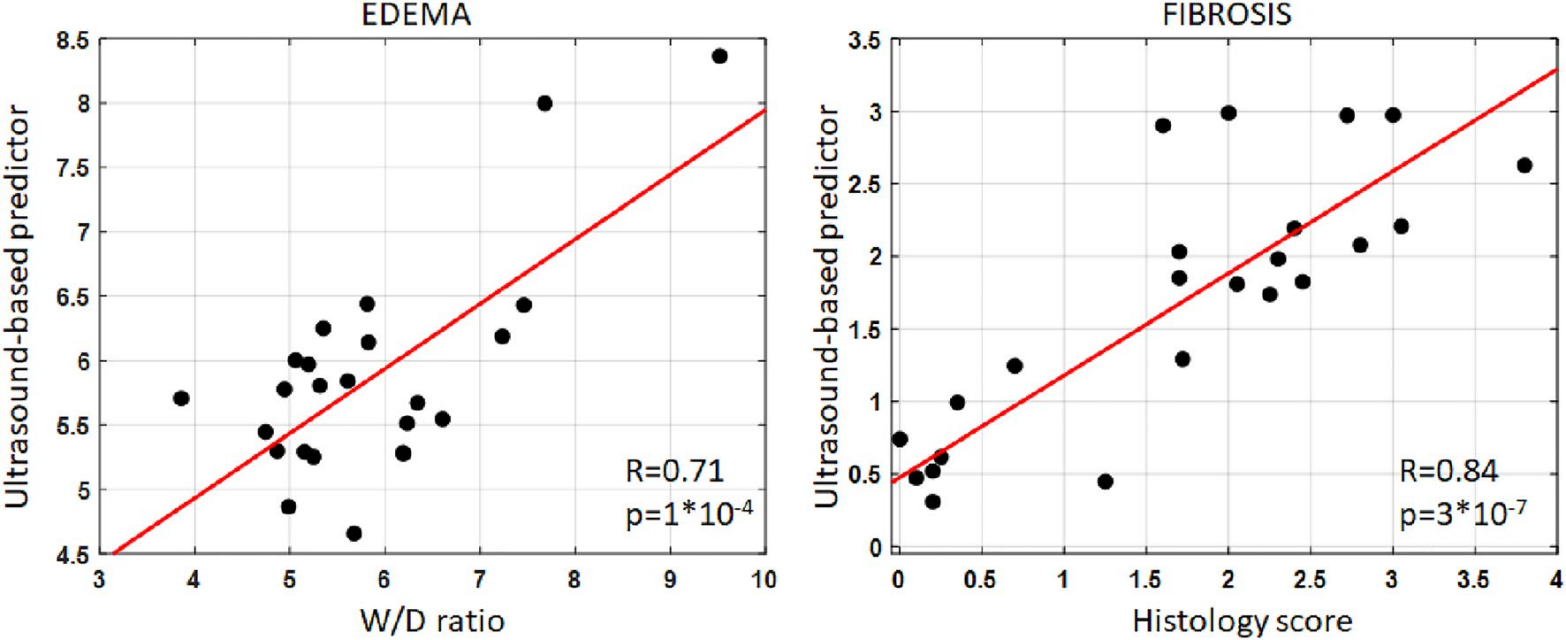
Regression plots of newly developed multilinear US based predictors combining multiple independent LQUS parameters, against severity of edema (W/D) and fibrosis (histology). Among the 60 measured LQUS parameters, combining BSC MF Slope and Nakagami m gave the best prediction of the severity of edema as measured by W/D (left). Combining SS_IDR, BSC Spectral Slope and the BSC MF Intercept gave the best prediction of the severity of edema as measured by histology (right).

A stepwise multilinear regression including all LQUS parameters demonstrates that combining three (for fibrosis) or two (for edema) LQUS parameters leads to ultrasound-based predictors of edema and fibrosis, with excellent correlations to the severity of edema (R = 0.71, *p* = 10^–4^) or fibrosis (R = 0.84, *p* = 3 * 10^–7^), as shown in Fig. 3. The ultrasound-based predictor of pulmonary edema was obtained by combining the BSC Midband Fit Slope (BSC MF_Slope) and the Nakagami shape parameter (Nakagami m). The ultrasound-based predictor of pulmonary fibrosis was obtained by combining the Single Scattering intensity decay rate (SS_IDR), the BSC Spectral Slope (BSC SS) and the BSC Midband Fit Intercept (BSC MF_I_0_).

### Specificity to edema and fibrosis

**Figure 4:**
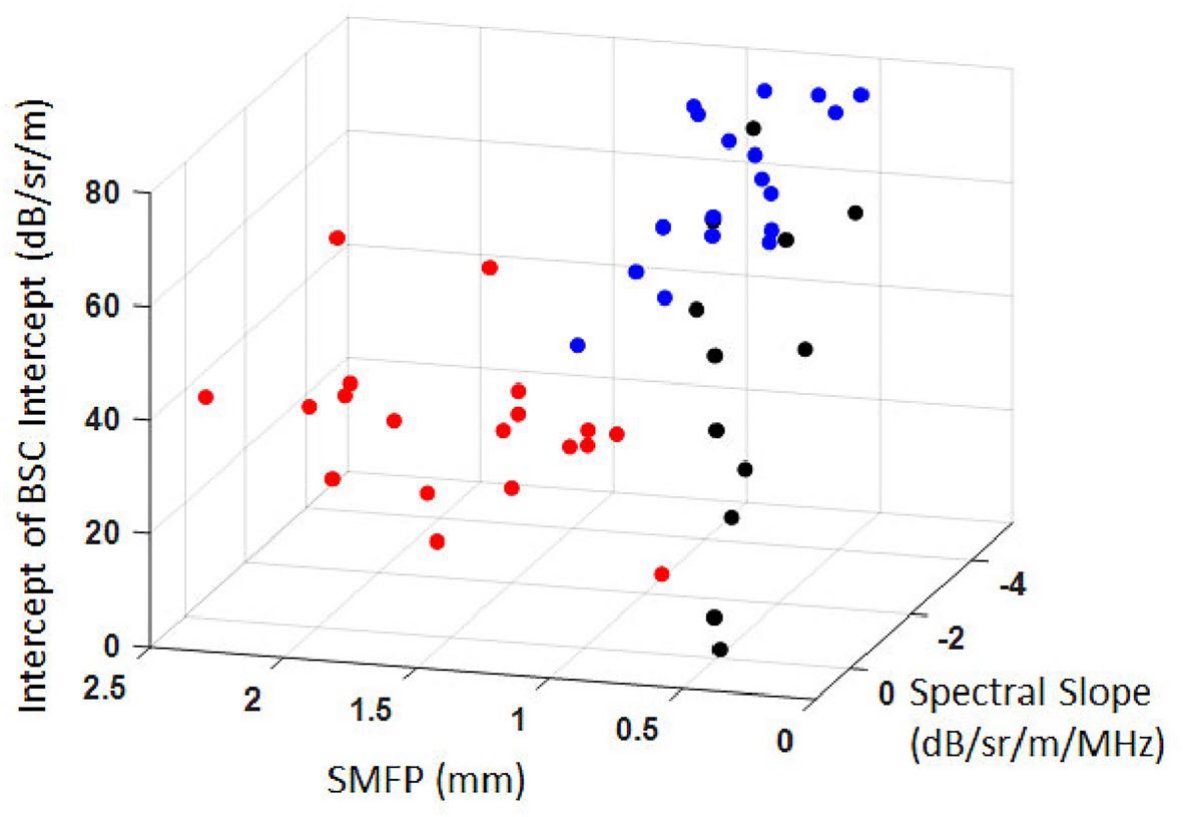
Combining SMFP, Intercept of BSC Intercept, BSC Spectral Slope allows to discriminate the control (black), fibrosis (red) and edema (blue) groups, to increase the specificity.

Measuring a large variety of LQUS parameters provides a detailed description of ultrasound scattering and of the lung microstructure. This large LQUS parameter space has the potential to provide sensitivity to various ILDs. From Table 1 and Fig. 3, it is encouraging to note that the most significantly contributing LQUS parameters are not the same for edema and fibrosis, suggesting that using specific combinations of LQUS parameters will ensure the specificity of each combination to different ILDs. As an example, Fig. 4 shows that in a parameter space comprised of SMFP (from the MS/SS family), the Midband Fit Intercept and the Spectral Slope (from the BSC family), control, fibrotic, and edematous lungs can be discriminated.

## Discussion

The accumulation of fluid in the alveoli and/or interstitium of edematous lungs, or the modification of the material properties of the alveolar walls in fibrotic lungs affect lung function by reducing volume of air within the alveoli. This reduction in air volume also alters the distribution of ultrasound scatterers throughout lung tissue, leading to an increased average distance between scatterers during propagation in lung tissue. It is through elegantly quantifying this phenomenon that LQUS parameters show promising potential as biomarkers for assessing pulmonary edema and fibrosis, allowing for the quantification of scatterer size and distribution within the lungs. Although not yet tested, this may be true for other forms of ILDs.

Individually, these LQUS parameters moderately but significantly (*p* < 0.05) correlated with the severity of edema or fibrosis with R values ranging from 0.50 to 0.61. When combined using a stepwise multilinear approach, large and highly significant correlations were obtained with the degree of severity of fibrosis (measured by histology), or edema (measured by W/D), because these LQUS parameters each reflect different features of the microstructure of lung tissue. The strength of the proposed method relies in the diversity of LQUS parameters measured, and the diversity of microstructural changes they quantify. For example, the BSC Midband Fit reflects the relative acoustic impedance between scatterers and the surrounding matrix, which in lungs will be sensitive to alveolar flooding. This is likely why this parameter is significantly different for fibrotic and edematous lungs, and significantly correlated to the severity of edema. As another example, changes in scatterer size leads to significant changes in the BSC Midband Fit, as well as in the BSC Midband Fit Spectral Slope. Larger alveolar wall thickness observed with fibrosis leads to larger distances between smaller air-filled alveoli on average. This leads to the larger SMFP values observed in fibrotic lungs. It can be expected that this diversity of parameters will enable LQUS methods to be sensitive and specific to various ILDs.

The analysis of ultrasound scattering in lung tissue holds significant potential as a routine monitoring modality, with distinct advantages over existing techniques for quantifying the severity of ILDs, such as CT scans. Combining LQUS parameters allows creation of quantitative biomarkers for lung fibrosis and edema, and potentially other ILDs. Utilizing LQUS to assess lung condition and follow response to treatment could revolutionize point-of-care lung evaluation. Now that there are effective therapies to reduce progression of fibrosis for idiopathic pulmonary fibrosis, and alterations in pulmonary edema due to treatments for heart failure, non-invasive point-of-care assessment based on interpretation of ultrasound data may become a useful clinical tool. Adopting a multi-parameter approach is particularly crucial for assessing lung health, as it allows for the differentiation of various pulmonary diseases and enhances the specificity of the quantitative method. The proposed LQUS approach will enable us to tailor the diagnosis and monitoring of many pulmonary diseases. Because the output of LQUS methods is quantitative measures of ultrasound-based predictors that will be easy to interpret by clinicians, adoption should be straightforward even in fast-paced clinical environments providing care for ILD patients or patients with heart failure.

These results were obtained with the ultrasound probe placed directly on the surface of the lung because the intercostal space in rats is too small to accommodate an ultrasound probe in the relevant frequency range. For clinical translation, it will be necessary to develop methods for measuring LQUS parameters through the chest wall. Because the insonification strategies required to measure the LQUS parameters rely on firing ultrasound pulses sequentially using single elements of a transducer array, developing novel strategies to account for and mitigate attenuation and diffraction effects in the chest wall will be necessary. Developing optimal transmit and beamforming sequences to be able to successfully measure LQUS parameters in vivo in humans through the chest wall will be the subject of a future study.

Linear regressions were used here because these simple classifiers are much less prone to overfitting that nonlinear classifiers which, in spite of the potential increased performance, were not used because of the current sample size and the risk of overfitting. It is remarkable that out of the many parameters significantly correlated to the severity of fibrosis or edema, only two parameters (BSC MF Slope and Nakagami m) were necessary to fit the severity of edema, and only three parameters (SS-IDR, BSC Spectral Slope and BSC MF Intercept) were necessary to fit the severity of fibrosis. The small number of parameters is a strong indication that the data was not overfit.

Combining different scattering based, BSC and EnvS LQUS parameters can improve detection and staging of pulmonary fibrosis and edema. Our results suggest that the combination of LQUS parameters could allow diagnosing ILDs with sensitivity and specificity. Such LQUS predictors will enable a convenient, non-ionizing method for diagnosis of pulmonary fibrosis, as well as provide a routine method to monitor severity of pulmonary fibrosis (indicating a potential need for performing a chest CT scan) and assessing severity of heart failure by quantifying severity of pulmonary edema.

## Materials and methods

### Surgical model of pulmonary edema

Rats were anesthetized with an intraperitoneal (ip) injection of ketamine (100 mg/kg) and xylazine (10 mg/kg), and intubated via tracheotomy through a cervical incision. Rats were ventilated with FiO2 1.0, tidal volume (TV) of 0.75 ml/100 gm body weight, rate 70/min, 3 cm H2O PEEP. Anesthesia was maintained with isoflurane (0.5–4%). Anesthesia depth was judged by toe pinch every 30 min. To maintain hydration, 1–2 cc sterile saline was injected subcutaneously (sc) each hour based on skin turgor and disappearance of the sc fluid. Animal temperature was monitored with a rectal probe and maintained at 37 °C on a heating pad intermittently turned on and off.

Pulmonary edema was induced in the left lung of rats using an established lung ischemia–reperfusion injury (IRI) model^15^. The anesthetized rat was placed in the right lateral decubitus position, and a left lateral thoracotomy was performed in the 4^th^ intercostal space. The left pulmonary hilum was dissected free. A small laparotomy incision exposed the liver. 600 U of heparin was injected intrahepatically. After 5 min, the left lung hilum was clamped with an aneurysm clip to render the left lung ischemic. The rat was placed in the supine position. To induce varying degrees of pulmonary edema, 3 different durations (20 (N = 6), 40 (N = 6), and 60 (N = 6) minutes) of lung ischemia were used before the clip was removed and the left lung reperfused for 60 min. Ultrasound data was acquired immediately after 60 min of reperfusion. As observed in^15^, pulmonary edema occurs immediately after the ischemia–reperfusion injury.

### Model of pulmonary fibrosis

After sedation (ketamine/xylazine as described above), rats were intubated with a 12-gauge catheter. Bleomycin 2 mg/kg, dissolved in 100 μl sterile phosphate buffered saline (PBS), was administered into the trachea^16^. The rat was extubated and allowed to recover. This bleomycin dose causes development of pulmonary fibrosis within 2–3 weeks. Rats were studied in groups of n = 6 (3 male, 3 female) 2, 3, and 4 weeks after bleomycin administration. This provided a range of severity of fibrosis for assessment. Six rats (3 male, 3 female) who received no treatment served as controls. Six more rats (3 males and 3 females) were treated with Nintedanib (10 mg/kg) administered daily by gavage after intra-tracheal bleomycin administration for 14 days. Nintedanib is an inhibitor of tyrosine kinase and is used in humans with idiopathic pulmonary fibrosis to delay progression of the disease^17^. Animal care was in accordance with institutional guidelines at the University of North Carolina at Chapel Hill, and all experiments were performed under Institutional Animal Care and Use Committee (IACUC) number 23-029.0 The UNC IACUC is under the oversight of the UNC Vice-Chancellor for Research.

### Surgery for ultrasound measurements, and euthanasia

After sedation (ketamine/xylazine as described above), a tracheotomy was performed. Rats were ventilated with a Harvard rodent ventilator. Anesthesia was maintained with titrated isoflurane (0.5–4%). A sternotomy was performed, both pleural spaces were opened, and the sternal edges spread maximally to expose both lungs. Hydration was maintained by subcutaneous administration of normal saline, 1–2 ml/h, based on skin turgor and fluid absorption. All rats were sacrificed by cardiectomy under anesthesia (a method of exsanguination under anesthesia, approved by the American Veterinary Medical Association). The incision was extended inferiorly into the abdomen to expose the liver. Heparin was administered intra-hepatically 5 min before sacrifice by incising the left and right atria to prevent clotting after the lungs are removed.

### Ultrasound data acquisition

A Verasonics (Kirkland, WA) Vantage 128 US scanner with a 7.8 MHz, 128-element linear array transducer (L11-4v) was used for in vivo data acquisition. A full synthetic aperture transmit sequence was used: each array element was excited individually, and the backscattered signals on all 128 elements were recorded for each transmit. This resulted in a 128 * 128 * t Inter-element Response Matrix (IRM), where *t* is the number of time points, with a 62.5 MHz sampling rate. Each of the 128 * 128 time trace was truncated into overlapping time windows T. LQUS parameters estimations have been presented in our previous studies^18^; they are briefly summarized below.

### Wet to dry ratio

Pulmonary edema was quantified by the wet/dry weight ratio (W/D). W/D was measured by excising the apical portion of the left lung, while the lung was in the chest with the rat in the supine position. This provided a combination of dependent (posterior) and non-dependent (anterior) lung tissue. Samples were placed on a previously weighed piece of aluminum foil, and each sample was weighed, then dried in an oven at 60 °C for 48 h, and weighed again. W/D is the ratio of the weights of the lung sample before and after desiccation and is an accepted method of assessing the amount of water in a lung sample^17,18^.

### Histology

Following the acquisition of ultrasound data, lung tissues were excised with the airway clamped at end-inspiration, and underwent inflation fixation by inflating them to the volume they appeared to be at when excised, then instilling 20 ml 10% paraformaldehyde through the main pulmonary artery from a height of 15 cm, followed by immersion in 10% paraformaldehyde for 24–48 h, Lung blocks were then placed in 70% ethanol. Subsequently, the lung blocks were embedded in paraffin and each lung was sectioned in the middle of the lung block from the apex to the base of the lung and 5 μm slices were obtained and mounted on glass slides. Sections were stained with hematoxylin and eosin (H&E), Sirius red special, and Masson’s Trichrome. Each lung was examined under a microscope at 100 × total magnification. For each lung, 10 different areas were selected for microscopic assessment, so 20 fields were averaged for each lung to create a score.

**Figure 5:**
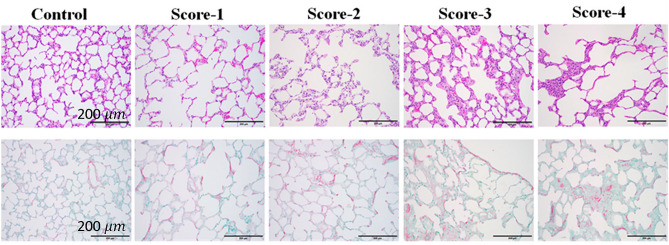
Examples of histology slides corresponding to modified Ashcroft scores 0–4, for H&E (top) and Masson’s trichrome (bottom) staining. Viewed at 100 × total magnification. Scale bar = 200 microns.

Severity of fibrosis was evaluated by assigning scores based on the modified Ashcroft scale^13,14^ by a veterinary pathologist (SM). A score of zero denoted completely healthy lung tissue (within normal limits), while a score of 8 indicated lung tissue field entirely affected by fibrosis. It should be noted that the maximum fibrosis score using the Ashcroft scale observed in this study was 4. Scores from 20 fields were then averaged to derive the final histology score for each lung (Fig. 5).

Histological examinations were carried out on both fibrotic and control lungs. Importantly, control lungs could be assigned a non-zero score (up to 0.25 on the modified Ashcroft fibrosis scale) due to a slight excess of normal collagen in some lung sections.

### Region of interest (ROI) selection

The signals were beamformed using the MUST (Matlab UltraSound Toolbox) platform^19^ in order to identify the pleural line in the lung and to manually choose the transducer elements that are above the lung (in some cases, the lung was smaller than the probe, and only the signals captured by the elements covering the lateral extent of the lung were selected). In propagation time, the signals were processed between the propagation time of the identified pleural line and a maximum propagation time of 13 μs, in order to ensure that only signals corresponding to lung tissue were evaluated (the rat lung at end expiration is approximately 10 mm thick). The ROI selection is illustrated in Fig. 1a–c of^18^.

### BSC-based LQUS parameter estimation

The mean magnitude squared of the Fourier transform of every US segment (i.e., transmit-receive pair over the windowed time) within each Region of Interest (ROI) was computed and log-compressed to derive an ROI spectrum, S_ROI_(f). The BSC is then computed using the reference phantom method^13,15^. S_REF_(f) is computed from the reference phantom using the same exact approach as in the lung ROI after compensation for the known attenuation in the reference phantom. The BSC of the ROI is then obtained by computing S_ROI_(f) − S_REF_(f) + BSC_REF_ where BSC_REF_ is the known BSC of the reference phantom. The BSC spectral slope, intercept (I0), and midband fit (MF) are computed in overlapping, rectangular time gates in each IRM by fitting the BSC by a straight-line. The average and standard deviation of each BSC-based LQUS parameter, as well as the slope and intercept of a linear function fit to each parameter over propagation time, were obtained (e.g., MF average, MF standard deviation, MF slope, and MF intercept were derived for MF). The estimation process of the BSC-based LQUS parameter is illustrated in Fig. 1d and f of^18^.

### EnvS-based LQUS parameter estimation

EnvS parameters based on the Nakagami and Homodyned-K distribution are computed in overlapping, rectangular time gates in each IRM using the same ROIs as the BSC-based LQUS parameters. The envelope of every RF segment in each ROI is computed by applying an Hilbert transform. Then, the probability density function (PDF) of the ROI is computed by assuming that each envelope value within the ROI is a random drawing from the underlying PDF. The Nakagami PDF was fit to the PDF of the ROI using a standard maximum likelihood estimator, yielding two LQUS parameters: the Nakagami shape parameter, m, and the Nakagami scale parameter Ω. The Homodyned-K PDF was fit to the PDF of the ROI using the standard XU estimator, which employs the first moment of envelope intensity and two log-moments to yield two LQUS parameters (Κ and β, Fig. 1 of^20^). The estimation process of the EnvS-based LQUS parameter is illustrated in Fig. 1e of^18^.

### Scattering Mean Free Path (SMFP)

SMFP represents the average distance between scatterers. It is expected to increase with edema due to increasing inter-alveoli distances. SMFP is obtained by extracting the incoherent intensity from the IRM and tracking the rate of growth of the incoherent halo over time^11^. Details of the method are available in^11^. Briefly, the SMFP is obtained through the measurement of the diffusion constant (D) via the following steps: An antisymmetric IRM is formed by taking the opposite of all values below the diagonal. The IRM and the antisymmetric IRM are truncated in 0.3 μs overlapping time windows (the duration of a time window is set to approximately two periods). A short-time Fourier transform is performed in each time window, and the backscattered intensity *I(X,T)* (from the IRM) and *I*^*A*^*(X,T)* (from the antisymmetric IRM) are obtained for each time window T and for each transmitter–receiver distance (X), by integrating the square of short-time Fourier transform. The incoherent contribution *I*_*inc*_*(X,T)* to the backscattered intensity is obtained by summing *I*^*A*^*(X,T)* and *I(X,T).* The diffusion constant is calculated by measuring the spatial growth of *I*_*inc*_*(X,T)* halo over time. SMFP is obtained from the diffusion constant (D) using: $SMFP=3D/c$ where c the estimated speed of sound (1500 m/s). The SMFP calculation method was improved over the method presented in^19^, by adding a region of interest selection step (see section on ROI selection above), to ensure only lung tissue is taken into account on the measurement. This is especially critical in rat lungs, which can be smaller than the footprint of the ultrasound probe.

### Singular value distributions measures (E and λ_max_)

*E* and λ_max_ are parameters related to the shape of the singular value distribution of the IRM. According to RMT, the distribution of singular values will resemble a quarter circle if MS dominates, and a Hankel function if single scattering (SS) dominates^21^. For each time window and at each frequency within the bandwidth, a singular value decomposition (SVD) is performed on the IRM. A histogram of the eigenvalues is computed and *E*, the expected value (area under the curve), and λ_max_, the value corresponding to the highest density of singular values were extracted. These two parameters relate to the shape of the singular value distribution and are therefore used to quantify the amount of MS in lung tissue. Because unhealthy lungs will create less multiple scattering, *E* is expected to be larger for unhealthy lungs, and λ_max_ is expected to be smaller.

### Separation of SS and MS contributions

The BSC and Env QUS methods described above are based on the assumption of weak and single scattering. In order to improve the sensitivity and specificity to lung pathologies of LQUS parameters, LQUS parameter estimation described above was performed on (1) the raw original IRM, (2) only the MS part of the IRM (MS-IRM), and (3) only the SS part of the IRM (SS-IRM). MS-IRM and SS-IRM are obtained using singular value decomposition based on RMT and on the fact that SS is responsible for a deterministic coherence along the anti-diagonal of the IRM. SVD is performed for each time window and the eigenvalues are obtained, as described above. An eigenvalue threshold is automatically determined as describe in^22^ and gives the rank of separation. According to RMT, the stronger eigenvalues correspond to the SS contribution and the weaker eigenvalues correspond to the MS contribution^23^. Once the SS and MS contributions are separated^24^, each LQUS parameter was obtained separately from the native IRM, the SS-IRM, and the MS-IRM (Fig. 1). This very comprehensive framework allows description of the microstructure of the lung tissues quantitatively and in great detail.

### Accounting for attenuation

As described above, for the BSC-based LQUS parameters, the known attenuation coefficient of the reference phantom was used to compensate attenuation from the reference phantom data. In the BSC- and Env-based LQUS parameter computations, no lung-attenuation compensation was performed. In addition to the loss of a linear relationship between propagation time and distance and the unknown attenuation value, the lung attenuation effect on the LQUS parameters was believed to be an important source of contrast between healthy and diseased lungs. Therefore, this additional quantitative source of contrast was used through its influence on the LQUS parameters. For the SMFP-based LQUS parameters, the growth of the diffusive halo over time is not affected by attenuation and attenuation compensation is not necessary.

### Frequency- and system-independence of BSC-based and Env-based LQUS parameters

The BSC estimated using the reference phantom method^13,15^ is a true tissue property and is not sensitive to the frequency used to excite the transducer, or even the scanner used, or scanner settings used as long as the reference phantom data was acquired with the same scanner, the same settings, and the same transmit frequency. Therefore, the BSC-based LQUS parameters are largely system and user independent^25^. (However, the BSC can only be estimated over the bandwidth providing sufficient signal-to-noise ratio, so it is possible that a different portion of the BSC will be estimated using a different transmit frequency or transducer.) Similarly, the PDF of the envelope of an ROI is associated with the number and position of the scatterers within the lung^25^ therefore, while the transmit frequency may affect the PDF it is unlikely to affect the LQUS parameters used here which were computed using the Homodyned-K and Nakagami distributions.

### Single scattering Intensity halo decay (i.e., SS_IDR)

**Figure 6:**
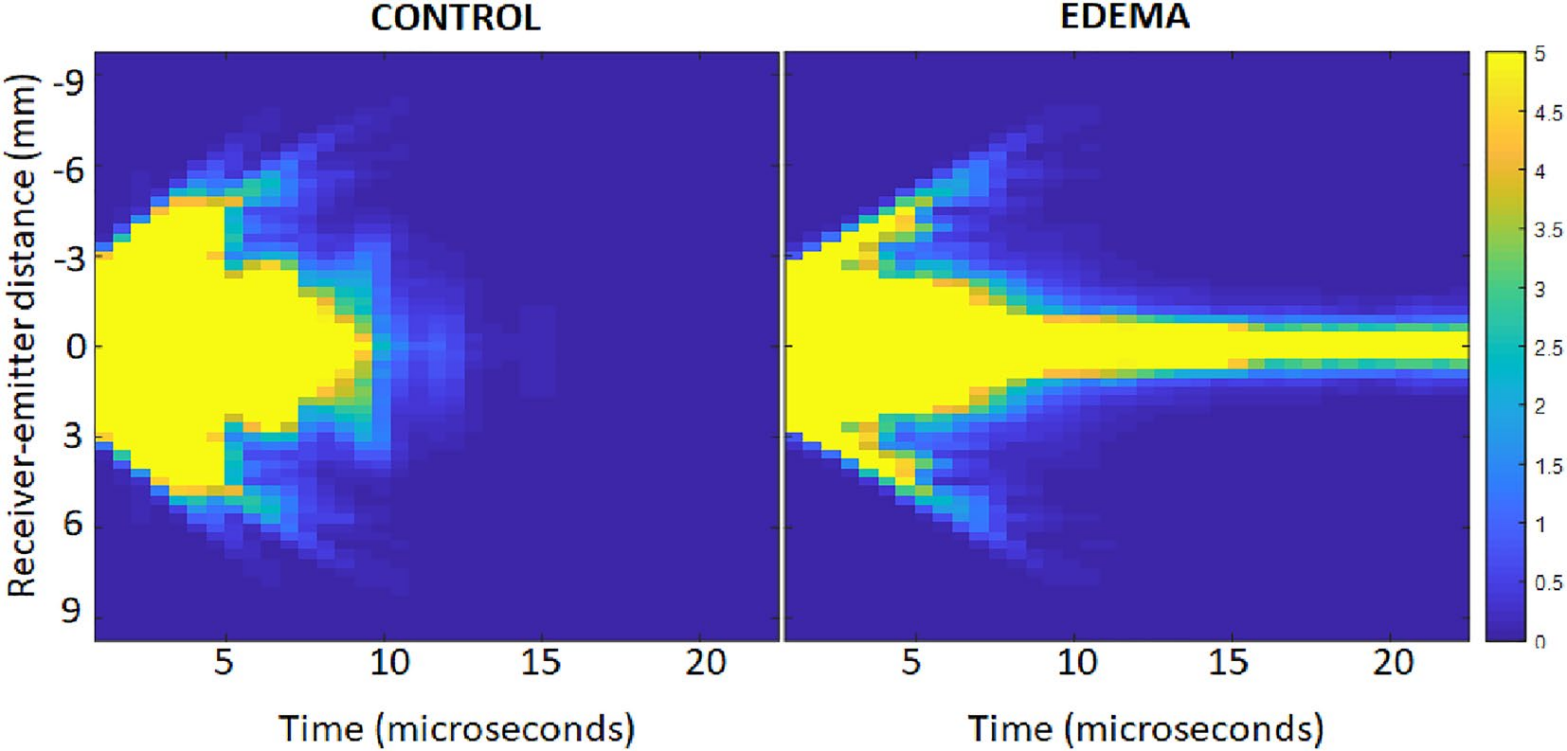
Single Scattering intensity halo as a function of time for a control lung (left) and for an edematous lung (right).

MS by alveoli should yield tremendous acoustic losses as a function of US propagation time. Figure 6 shows the acoustic intensity halo *I(r,T)* (i.e., the square of the US signals summed over time for overlapping time windows) as a function of time T in a control rat lung (left) and in an edematous rat lung (right). *r* is the emitter-receiver distance. The width of *I(r,T)* decays much faster in the control lung than in the edematous lung (Fig. 6). This decay is quantified by fitting a Gaussian curve to *I(r,T)* for each time window.

## Data Availability

The data that support the findings of this study are available from one of the corresponding authors, MM, upon request. The data consist of raw ultrasound data, as well as wet to dry weight ratios (for edematous lungs) and histology images (for fibrotic lungs).
